# Electroconvulsive therapy: improved understanding of long-term risks and benefits from advances in administrative health data

**DOI:** 10.1192/bjp.2026.10613

**Published:** 2026-04-16

**Authors:** Tyler S. Kaster, Taeho Greg Rhee, Erin Adler, George Kirov

**Affiliations:** **Tyler S. Kaster**, Temerty Centre for Therapeutic Brain Intervention, Campbell Family Mental Health Research Institute, Centre for Addiction and Mental Health, Toronto, Ontario, Canada; Department of Psychiatry, University of Toronto, Toronto, Ontario, Canada; Campbell Family Mental Health Research Institute, Centre for Addiction and Mental Health, Toronto, Ontario, Canada; Institute of Health Policy, Management and Evaluation, University of Toronto, Toronto, Ontario, Canada; and ICES, Toronto, Ontario, Canada; **Taeho Greg Rhee**, Department of Psychiatry, Yale University School of Medicine, New Haven, Connecticut, USA; New England Mental Illness, Research Education, and Clinical Center, VA Connecticut Healthcare System, West Haven, Connecticut, USA; and Department of Public Health Sciences, University of Connecticut School of Medicine, Farmington, Connecticut, USA; **Erin Adler**, Temerty Centre for Therapeutic Brain Intervention, Campbell Family Mental Health Research Institute, Centre for Addiction and Mental Health, Toronto, Ontario, Canada; **George Kirov**, Division of Psychological Medicine and Clinical Neuroscience, Cardiff University, Cardiff, UK

**Keywords:** Electroconvulsive therapy, mortality and morbidity, dementias/neurodegenerative diseases, population-level, administrative data

## Abstract

Electroconvulsive therapy (ECT) is an established intervention for severe or treatment-resistant psychiatric illnesses, including depression, schizophrenia, mania and catatonia. Despite its efficacy, concerns over its risks have contributed to ongoing stigma and hesitancy regarding its use. Traditional clinical trials have demonstrated the superiority of ECT in symptom reduction compared with other treatments, yet are impractical for assessing rare or long-term outcomes. Observational studies using administrative health data can assess rare or long-term outcomes, but are limited by confounding/bias. This review synthesises evidence from studies utilising administrative health data and modern statistical methods to address clinically relevant questions about ECT’s association with (a) dementia, (b) major adverse cardiovascular/cerebrovascular events, (c) suicide deaths and (d) all-cause mortality. Most studies indicate that, after adjusting for confounding, ECT does not increase the risk of dementia or major adverse cardiovascular/cerebrovascular events. Furthermore, ECT is likely associated with a substantial reduction in suicide mortality and all-cause mortality. Although observational studies cannot fully eliminate unmeasured confounding, the consistency of the findings from diverse investigators and congruence with clinical trials and neuroimaging studies lends support to their validity. These modern observational studies have yielded results that reinforce ECT’s safety and efficacy supporting its position as a life-saving treatment.

## Electroconvulsive therapy

### Background

Electroconvulsive therapy (ECT) is an established intervention for severe or treatment-resistant psychiatric illnesses, including depression, schizophrenia, mania and catatonia.^1^ It involves the administration of a repetitive electrical stimulus to the head of the patient, to cause a generalised tonic-clonic seizure after an individual has received a general anaesthetic and muscle relaxant. For individuals with severe or treatment-refractory depression, it can achieve remission or resolution of symptoms in up to 60% of individuals, although even higher rates have been reported.^2^ It also may be particularly effective for individuals with depression and associated psychotic symptoms or suicidality.^3^ Similarly, for individuals with schizophrenia experiencing refractory psychotic symptoms that have not responded to clozapine, ECT can significantly reduce psychotic symptoms in half of these individuals.^3^ ECT is also extremely effective for treatment-resistant mania and catatonia, both of which can be associated with life-threatening consequences.^3^ Because of its critical role in treatment-resistant psychiatric illnesses, ECT is recognised as an important treatment by professional practice societies around the world, and is recommended by the National Institute of Health and Care Excellence.^4^

### Known risks

Although ECT has well-established evidence of efficacy for alleviating the symptoms of severe or treatment-resistant psychiatric illness,^2^ the known risks and side-effects of ECT are a key consideration of this treatment and a source of stigma and resistance to it. The most prominent of these are the cognitive side-effects that can occur with treatment and are often a particular source of concern for patients. Furthermore, given that ECT involves a general anaesthetic and muscle relaxant and is accompanied by increases in heart rate and blood pressure,^3^ other side-effects can also result from the treatment, including myalgias, headaches, aspiration, arrhythmias and, in the rarest cases, even death.^3^ Concerns surrounding these potential side-effects, especially in frail or older adults, are often the most important consideration – rather than concerns of symptom reduction – when an individual (or their loved ones) is considering ECT treatment.

In the process of considering ECT treatment, the informed decision-making process is critically important, given the treatment’s history and societal perspective. Most of our knowledge regarding the risks and benefits of ECT comes from small-scale observational studies or clinical trials that examine symptoms from questionnaires or examine common outcomes such as myalgias or cognitive effects.^2^ Although these study designs provide important information, they cannot answer questions pertaining to long-term outcomes – such as dementia or premature mortality – that many patients most want to understand.

### Limits of clinical trials

Randomised clinical trials (RCTs) have been fundamental to the advance of medicine. This is due to the importance of randomisation, in which participants are randomly assigned to groups by chance, which allows for unbiased estimates of treatment effects. As it pertains to ECT, RCTs have yielded decisive evidence regarding the efficacy of ECT treatment, as highlighted by the seminal meta-analysis RCTs of ECT published more than two decades ago by the UK ECT Review Group.^2^

This meta-analysis found that active compared with sham ECT reduced depressive symptoms by a standardised mean difference (SMD) of 0.91 (approximately 9.7 points on the Hamilton Rating Scale for Depression) in favour of active ECT.^2^ Similarly, active ECT compared with pharmacotherapy reduced depressive symptoms by an SMD of 1.01.^2^ A more recent network meta-analysis of non-surgical brain stimulation for depression found that ECT was superior to all other forms of brain stimulation, including repetitive transcranial magnetic stimulation, magnetic seizure therapy and transcranial direct current stimulation.^5^ Collectively, these results from clinical trials have demonstrated a convincing effect of ECT on reducing depressive symptoms superior to other treatment modalities.

Although these findings have informed clinical care and patient decision-making, the limits of RCTs become apparent when examining relationships of interventions with rare events such as adverse medical events, suicide deaths and all-cause mortality. The rarity of these outcomes means that thousands of participants would be required for sufficient statistical power – an impractical size for most trials. Even greater difficulties arise attempting to assess longer-term outcomes that may occur years or decades later, given that the longest duration RCT included in the UK ECT Review Group meta-analysis was 12 weeks.^2^ Furthermore, individuals participating in clinical trials represent a small subset of the individuals receiving care in real-world clinical settings, sometimes as little as 20%.^6^

Because clinical trials cannot capture rare or long-term outcomes, or may not reflect typical patients receiving treatment, important gaps remain in understanding the risks and benefits of ECT. Answering these types of questions requires an approach fundamentally different from clinical trials in which large, real-world data-sets with long follow-up are analysed using modern statistical methods designed to overcome the limits of observational research. Recent advances in both these domains have enabled a fundamentally new approach for generating evidence to improve the shared decision-making process for patients and clinicians considering ECT.

## Administrative health research to inform clinical care

### Administrative health data

Organisations administering healthcare require records of their administrative activity. These organisations – hospitals, health insurers and health maintenance organisations – store these records in databases that can then be reviewed and used to inform their ongoing activities. With the development of computerised databases, the availability of these data has surged in the past number of years.^7^ The data can include information on records of health services, diagnostic/medical procedures, prescription information and diagnostic information.^7^ Although collected for administrative purposes, this information has increasingly been used as a data source for research.^7^

An advantage of administrative health data for research is that it contains large numbers of individuals such as the entire population of a geographic region/country, which may include the health information for several million individuals.^7^ Furthermore, these data – particularly when collected at the population level – provide a more representative sample in real-world settings compared with RCTs, which may include restrictive eligibility criteria. As a result, clinical trial results may not apply to the target treatment population.^8^ In contrast, administrative health data allows for assessing study outcomes of an intervention directly within the target population.

Administrative health data offer important strengths, but two major limitations exist. First, because these data are collected for healthcare administration rather than research, investigators cannot choose which variables are captured and must therefore shape questions around available data. Second, particularly when considering interventions, the researcher does not allocate individuals to an intervention. As a result confounding – especially confounding by indication – can occur when assessing associations between an intervention and an outcome.^8^ With ECT, many of the characteristics associated with receiving ECT (e.g. suicidality, psychosis and depression severity) are associated with negative outcomes.^9^ Given these limitations, the methods used in the analysis of administrative health data must minimise biases to produce valid estimates of intervention effects.

### Methodological developments

In the past two decades, there has been increased awareness of statistical methods and frameworks to mitigate confounding that occurs when estimating treatment effects from observational data. It is now recognised that administrative health data can inform clinical care and serve as the basis for regulatory approvals as highlighted by the US Food and Drug Administration’s Real World Evidence Program.^10^ When observational studies are designed with care, for example by using a target trial framework,^11^ it is possible to develop treatment effect estimates where bias is sufficiently minimised such that it can inform clinical decision-making.

Although there are various methods for minimising confounding in observational studies, the most common approach is the use of regression-based models. In these models, the investigator will specify an outcome variable (e.g. hospital admission), an exposure variable (e.g. ECT exposure) and then ‘adjust’ for confounders, which are characteristics associated with both the exposure and outcome, but not along the causal pathway.^8^ An important limitation of this approach is that, distinct from RCTs, this approach combines the ‘design’ and ‘analysis’ phase such that researchers may be tempted to modify their model until suitable results are obtained.^8^

One approach that has recently gained popularity is the use of propensity scores, which are the probability an individual is exposed to an intervention based on their pre-exposure characteristics.^8^ Subject to several important assumptions, when propensity scores are used to ‘balance’ patient characteristics in a study sample at baseline, any differences in outcomes can then be attributed to the intervention.^8^ This approach separates the design and analysis phase similar to a clinical trial. For example, a researcher may iteratively modify the propensity score until adequate balance is achieved on all baseline observed characteristics (design) before performing the primary study analysis of the outcome. Although propensity scores are useful tools, one limitation is that they only account for confounding captured in variables available in the data-set such that unobserved confounding remains a possibility.^8^ However, to the extent that unobserved covariates correlate with observed variables (e.g. medical comorbidities are correlated with age), then propensity scores and adjustment can mitigate the effect of these confounders even if unobserved.^8^

Despite these limitations, rigorous application of advanced methods to large administrative databases can yield new insights into the long-term risks and benefits of ECT, providing answers to clinically important questions that cannot be addressed in clinical trials.

## ECT questions addressed with administrative health data

### Approach

We conducted a systematic search of Medline on 9 October 2025, using keywords for electroconvulsive therapy and observational/cohort studies from 2010 to present, using the Ovid interface (Supplementary Material). Studies before 2010 were excluded because they were less likely to have used modern statistical methods to address confounding and/or have access to comprehensive administrative health databases. We included any comparative study using population-level administrative health data to examine the following outcomes: (a) dementia, (b) major adverse cardiovascular/cerebrovascular events, (c) suicide deaths and (d) all-cause mortality.

We used a critical lens in reviewing these studies, but not a formal meta-analytic or risk of bias framework in examining them, as the intent of this work is to provide an up-to-date summary of recent studies focusing only on administrative health data to answer clinically relevant questions related to ECT outcomes. However, we did separately include any relevant meta-analyses previously reporting on these outcomes. We also cross-referenced the articles included in the meta-analyses with those from our systematic search, to minimise the risk of missing relevant articles.

### ECT and dementia

Although imaging evidence does not support any indication of neurological damage associated with ECT,^12^ given the known cognitive side-effects that occur with ECT treatment (e.g. anterograde/retrograde amnesia or subjective cognitive complaints),^13^ a common concern for patients is the eventual development of dementia. Administrative health data is poorly suited to quantifying the nuanced cognitive side-effects related to ECT, but it is an ideal approach for identifying any potential association between ECT and subsequent development of dementia, years or even decades after treatment. Quantifying this association is particularly challenging given that the indication for ECT treatment itself (i.e. depression) is a known risk factor for developing dementia.^14^

**Fig. 1:**
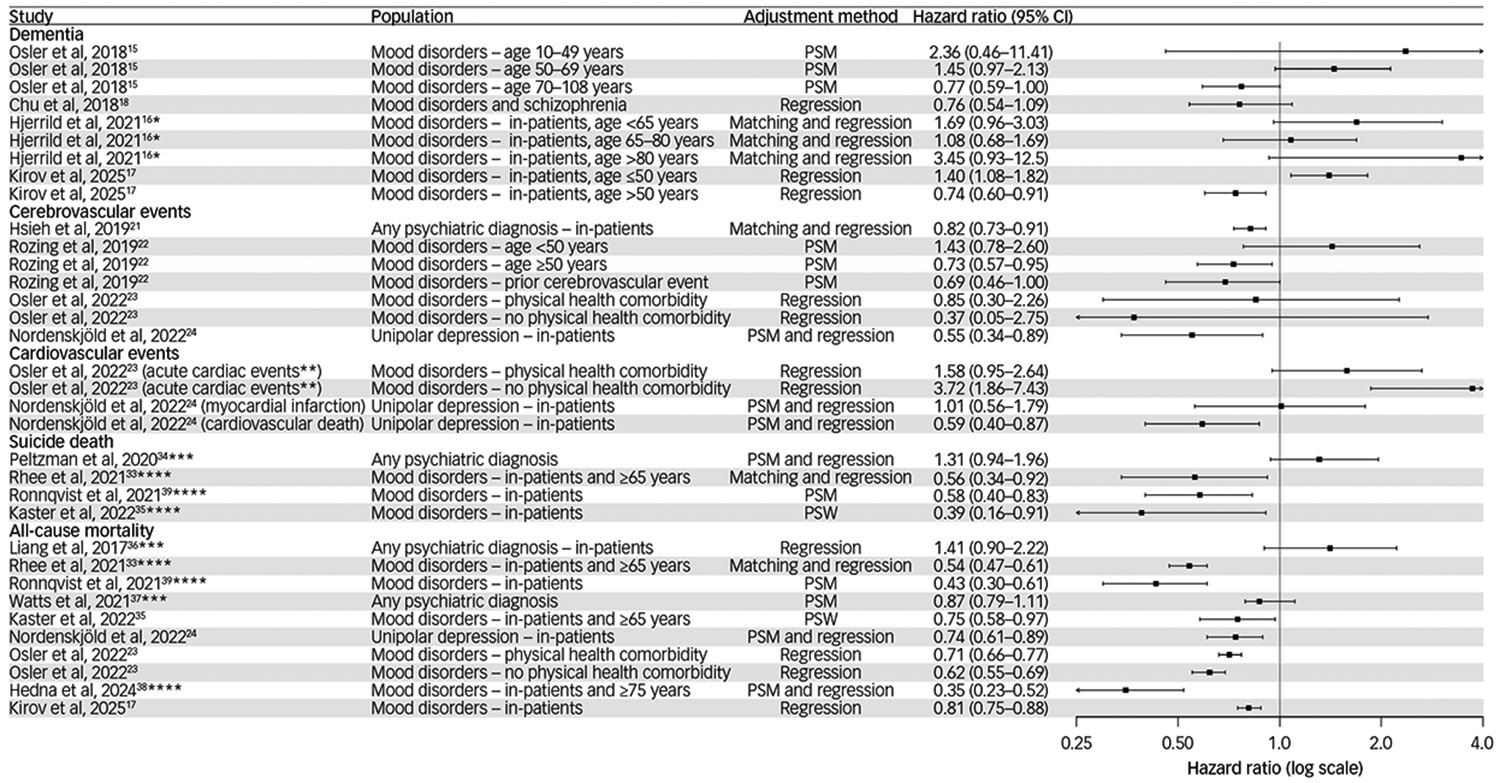
Association of electroconvulsive therapy with dementia, major adverse cardiovascular/cerebrovascular events, suicide deaths and all-cause mortality. *Point estimate and confidence interval were inverted from original publication to use no electroconvulsive therapy exposure as reference group. **Acute cardiac events: acute myocardial infarction, cardiac arrest, paroxysmal tachycardia, atrial fibrillation/flutter and other cardiac arrhythmias. ***Reported odds ratio. ****90-day outcome. Note: Ahmadi et al,^26^ Liang et al^32^ and Jørgensen et al^31^ are not included in this figure because of methodological limitations. PSM, propensity score matching; PSW, propensity score weighting.

Our systematic search identified four studies examining the association between ECT and dementia and no systematic reviews (Fig. 1). Several of these studies found that before confounding was considered, there was a higher incidence of dementia among those receiving ECT versus not receiving ECT.^15,16^ However, once confounding was accounted for all studies found that there was no indication ECT increased the risk of developing dementia, except for the younger age subgroup in one study, which had few events and a small sample size.^17^ This was the case both in the short^18^ and long term,^16,17^ and among those with schizophrenia.^18^

### ECT and major adverse cardiovascular/cerebrovascular events

Administering ECT results in a series of alternating parasympathetic and sympathetic events. The electrical stimulus causes a parasympathetic reaction through indirect electrical stimulation of the vagal nerve,^19^ which is followed by increased sympathetic activity when the seizure occurs, and finally, upon seizure termination, there is once again increased parasympathetic activity.^20^ In addition to the physiological effects of ECT, there can also be haemodynamic consequences of the general anaesthetics used for ECT.^3^ As a result, patients and physicians are often concerned as to whether ECT significantly increases the risk of major adverse cardiovascular or cerebrovascular events.

Our systematic search identified two studies examining cerebrovascular events, two studies examining cardiovascular outcomes, two studies examining both (Fig. 1) and one relevant systematic review. Regarding the risk of ECT associated with cerebrovascular events, there did not appear to be any increased risk associated with ECT for both new and recurrent cerebrovascular events^21,22^ or in those with pre-existing physical health conditions.^23^ Although three studies suggested a reduced risk associated with ECT,^21,22,24^ a sensitivity analysis that used the Fine and Gray method^8^ to account for competing risks found that there was no association (subdistribution hazard ratio (sdHR): 0.86, 95% CI 0.67–1.10) between ECT and risk of incident stroke, suggesting the observed reduction may be attributable to the competing risk of mortality.^22^

For the risk of ECT associated with major adverse cardiovascular events, the evidence is more conflicting (Fig. 1). The systematic review examining the association between ECT and major adverse cardiovascular events reported the incidence of cardiac events to be quite low (i.e. one in 50 patients);^25^ however, this meta-analysis included studies without comparator groups such that these meta-analytic results cannot be used to draw inferences about the effect of ECT on these outcomes.

In terms of comparative studies, one of the first studies used data from the Department of Veterans Affairs in the USA and found a significant reduction in cardiovascular mortality for individuals with post-traumatic stress disorder and major depressive disorder receiving ECT compared with not receiving ECT.^26^ However, this study had several methodological limitations that hinder the interpretability of these results and was excluded from our reporting (Supplementary Material). A more recent study found that, among individuals with mood disorders who had physical comorbidities, there was no significantly increased risk of major adverse cardiovascular event after ECT.^23^ Interestingly, those without physical comorbidities did have a significantly increased risk of major adverse cardiovascular event after ECT, although the authors indicate this was based on very few events and the increased risk did not persist beyond 30 days.^23^ In the only study conducted to date that focused on major adverse cardiovascular events and ECT and used propensity score matching and regression adjustment, the authors found that individuals with affective disorders who received ECT did not have an increased risk of myocardial infarction, but did have a reduced risk of cardiovascular death in the year following admission. No study used a Fine and Gray model for competing risk.

### ECT and suicide deaths

One of the main indications for ECT is treatment of psychiatric conditions (most commonly severe depression) in the presence of acute or severe suicidal ideation.^3^ ECT has been associated with both substantial and rapid reduction of suicidal thoughts, which has often been assumed to translate to reduction in suicide death.^1^ However, one of the challenges with testing this assertion is that the severity of illness for those receiving ECT is often such that they are at greater risk of dying from suicide entirely independent from ECT.^9^ Therefore, determining whether the symptom reduction observed with ECT is associated with reduced suicide deaths is one of the most critical questions related to ECT.

Our search identified four relevant systematic reviews, all of which were published in 2025. These studies reported a significant association between ECT and reduced risk of suicide death in the range of 30–50%.^27-29^ One meta-analysis did report discrepant results, with no association identified between ECT and suicide deaths^30^ and another demonstrating heterogeneity regarding the association between ECT and suicide deaths based on follow-up time and geographic region.^27^ Of note, the one meta-analysis that also examined the effect of antidepressant treatment (versus no treatment) found that there was no association between antidepressant treatment and the risk of suicide deaths.^28^

In our systematic search, we identified eight population-level studies examining the association between suicide deaths (Fig. 1) that, consistent with the systematic reviews, typically identified a significant reduction in suicide-related mortality associated with ECT. Similar to other outcomes, we did not include the study by Ahmadi et al because of its methodological limitations.^26^ Also of note is that we did not include the study by Jørgensen et al, as the design of this study likely results in bias of the effect of ECT on suicide deaths (Supplementary Material).^31^ We similarly excluded Liang et al because of study methodological limitations and the resulting risk of bias (Supplementary Material).^32^ Although most studies did identify a significantly reduced risk of suicide mortality associated with ECT, there was some variability in these findings in that longer follow-up periods (e.g. a year after ECT) were not always associated with reductions in suicide death,^33,34^ but this was not the case for all studies.^35^ Some of this variability in findings may be attributable to not fully accounting for differences between ECT and non-ECT groups (despite propensity score matching) on important characteristics such as illness severity or pharmacotherapy,^34^ or differences in past number of hospital admission.^32^ Despite these limitations, no study – with the exception of Jørgensen et al^31^ – identified a significantly increased risk of suicide death once confounding was addressed.

### ECT and all-cause mortality

The single most important metric to determine whether ECT is a ‘life-saving’ procedure as has been claimed^1^ is the rate of all-cause mortality, which is a single metric that encompasses both the risks and benefits of ECT.

Our search identified four systematic reviews that are the same reviews as those for suicide death, which often also consider all-cause mortality. Each one yielded a remarkably consistent 25–30% reduction in all-cause mortality associated with ECT despite differences in analytic techniques.^27-30^ This is a particularly important finding as all-cause mortality is not susceptible to competing risk or diagnostic misclassification, which can arise in the context of cause-specific mortality.^8^

Regarding specific studies assessing all-cause mortality, our search identified 11 population-level studies assessing the association between all-cause mortality and ECT. Similar to other outcomes, the studies by Ahmadi et al and Jørgensen et al were not included.^26,31^ Studies assessing the risk of all-cause mortality have consistently found a reduced risk of death associated with ECT and have come from regions including Taiwan,^36^ USA,^33,37^ Canada,^35^ Denmark,^23,31^ Sweden^38,39^ and the UK.^17^ Although it is possible these findings may be related to confounding (i.e. healthier individuals receive ECT), differences in healthcare systems or access to ECT, the multitude of investigators in different populations, with distinct and rigorous methods, finding similar results lends greater support to the position that ECT may be a life-saving procedure. The potential mechanism for this – rather than due to fewer medical comorbidities among those receiving ECT, which is typically accounted for – is that given its significant reduction in suicide death, combined with a benign medical profile,^40^ ECT treatment may result in a net reduction in all-cause mortality for those with severe psychiatric illness.

## Advancing shared decision-making for ECT

This work highlights a number of high-quality, population-level studies examining the association between ECT and relevant clinical outcomes that cannot be adequately assessed in clinical trials. Although this was not a formal meta-analysis, the findings from many independent investigators in different populations, using different methods, collectively reinforce what is known by many clinicians – that ECT is a safe and effective procedure. ECT does not increase the risk of dementia or major adverse cardiovascular/cerebrovascular events, yet it significantly reduces suicide deaths to the point that it may result in a reduction of all-cause mortality, providing empirical support for the claim that ECT is a ‘life-saving procedure’.

Observational studies can never fully eliminate the possibility of unobserved confounding. Yet clinical trials suffer from inherent limitations that can never be overcome, such as the need for an individual to meet the eligibility criteria, agree to participate in the series of assessments or agree to the process of randomisation. These limitations inherently limit the generalisability of clinical trials and often exclude the most clinically unwell individuals who may stand to have the greatest benefit from ECT. The findings of the observational studies highlighted in this work are both consistent with the clinical trials of ECT that demonstrate significant reductions in suicidal thoughts^1^ and with neuroimaging studies that fail to find any evidence of post-ECT brain gliosis or oedema (with some indication that grey matter volume is increased post-ECT).^12^ Yet they extend these findings by looking at the real-world outcomes that matter to patients, such as suicide death or the development of dementia.

Despite these advances, there are still important knowledge gaps in the use of ECT in a clinically representative population. Most of these studies considered depression as the primary indication for ECT, or at most included a mixed diagnostic sample. However, dedicated studies of ECT-responsive conditions such as schizophrenia, catatonia or bipolar mania that use observational data have yet to be conducted. Similarly, future work would benefit from the integration of the clinical trial approach with administrative health data; for example, by conducting an RCT linking participant data to administrative health databases to collect long-term outcomes on larger numbers of participants.

The integration of administrative health data with modern statistical methods – much as early clinical trials transformed medicine – has begun to advance our understanding of rare and long-term outcomes. When rigorously conducted, observational studies can meaningfully inform shared decision-making. For ECT, this approach has clarified risks and benefits beyond the reach of clinical trials and reaffirmed that ECT is a life-saving, essential treatment for severe psychiatric illness.

## Supplementary Material

Supplemental Materials

The supplementary material is available online at https://doi.org/10.1192/bjp.2026.10613

## Data Availability

Data is available upon request from the corresponding author, T.S.K.
