# Supplementary material for: Electroconvulsive therapy: improved understanding of long-term risks and benefits from advances in administrative health data
Source: Br J Psychiatry. Author manuscript; Available in PMC 2026 May 14. (PMC13173538; doi:10.1192/bjp.2026.10613)
Supplement: Supplemental Materials [file NIHMS2166886-supplement-Supplemental_Materials.docx]

Table of Contents

[Supplemental Table 1 – Search Strategy 2](#_Toc220400167)

[Supplemental Table 2 – Studies examining association between ECT and development of dementia 3](#_Toc220400168)

[Supplemental Table 3 – Studies examining association between ECT and cerebrovascular events 3](#_Toc220400169)

[Supplemental Table 4 – Studies examining association between ECT and cardiovascular events 4](#_Toc220400170)

[Supplemental Table 5 – Studies examining association between ECT and suicide death 4](#_Toc220400171)

[Supplemental Table 6 – Studies examining association between ECT and all-cause mortality 5](#_Toc220400172)

[Supplemental Table 7 – Methodologic Limitations of Excluded Studies 7](#_Toc220400173)

[Ahmadi et al. 2016 7](#_Toc220400174)

[Liang et al. 2018 7](#_Toc220400175)

[Jorgensen et al. 2020 8](#_Toc220400176)

[Supplemental Table 8 – Study characteristics 9](#_Toc220400177)

[Supplemental Figure 1 – PRISMA Diagram 23](#_Toc220400178)

[Supplemental References 24](#_Toc220400179)

# Supplemental Table 1 – Search Strategy

Search was conducted on October 9, 2025

| 1 | exp Convulsive Therapy/ |
| --- | --- |
| 2 | convulsive therapy.mp. |
| 3 | exp Electroconvulsive Therapy/ |
| 4 | electroconvulsive therapy.mp. |
| 5 | 1 or 2 or 3 or 4 |
| 6 | exp Observational Study/ |
| 7 | observational stud*.mp. |
| 8 | exp Cohort Studies/ |
| 9 | cohort stud*.mp. |
| 10 | 6 or 7 or 8 or 9 |
| 11 | 5 and 10 |
| 12 | limit 11 to yr="2010 -Current" |

# Supplemental Table 2 – Studies examining association between ECT and development of dementia

| **Study** | **Population** | **Adjustment Method** | **HR (95%CI)** |
| --- | --- | --- | --- |
| Osler et al. 2018(1) | Mood disorders – Age 10-49 | Propensity score matching | 2.36 (0.46–11.41) |
| Osler et al. 2018(1) | Mood disorders – Age 50-69 | Propensity score matching | 1.45 (0.97–2.13) |
| Osler et al. 2018(1) | Mood disorders – Age 70-108 | Propensity score matching | 0.77 (0.59–1.00) |
| Chu et al. 2018(2) | Mood disorders and schizophrenia | Regression model | 0.76 (0.54-1.09) |
| Hjerrild et al. 2021*(3) | Inpatients with mood disorders | Matching and regression model | 1.37 (0.95-1.92) |
| Kirov et al. 2025(4) | Inpatients with mood disorders | Regression model | 0.89 (0.76-1.04) |

HR – Hazard ratio; CI – Confidence Interval

*point estimate and confidence interval were inverted from original publication to use non-ECT exposed as reference group

# Supplemental Table 3 – Studies examining association between ECT and cerebrovascular events

| **Study** | **Population** | **Adjustment Method** | **HR (95%CI)** |
| --- | --- | --- | --- |
| Hsieh et al. 2019(5) | Inpatient with any psychiatric illness | Matching and regression analysis | 0.82 (0.73-0.91) |
| Rozing et al. 2019(6) | Mood disorder – age <50 | Propensity score matching | 1.43 (0.78-2.60) |
| Rozing et al. 2019(6) | Mood disorder – age ≥50 | Propensity score matching | 0.73 (0.57-0.95) |
| Rozing et al. 2019(6) | Mood disorder – Prior cerebrovascular event | Propensity score matching | 0.69 (0.46-1.00) |
| Osler et al. 2022(7) | Mood disorder –physical health comorbidity | Regression analysis | 0.85 (0.30–2.26) |
| Osler et al. 2022(7) | Mood disorder –no physical health comorbidity | Regression analysis | 0.37 (0.05–2.75) |
| Nordenskjöld et al. 2022(8) | Inpatients with unipolar depression | Propensity score matching and regression analysis | 0.55 (0.34–0.89) |

HR – Hazard ratio; CI – Confidence Interval

# Supplemental Table 4 – Studies examining association between ECT and cardiovascular events

| **Study** | **Population** | **Adjustment Method** | **Outcome** | **HR (95%CI)** |
| --- | --- | --- | --- | --- |
| Osler et al. 2022(7) | Mood disorder –physical health comorbidity | Regression analysis | Acute cardiac events* | 1.58 (0.95–2.64) |
| Osler et al. 2022(7) | Mood disorder –no physical health comorbidity | Regression analysis | Acute cardiac events* | 3.72 (1.86–7.43) |
| Nordenskjöld et al. 2022(8) | Inpatients with unipolar depression | Propensity score matching and regression analysis | Myocardial infarction | 1.01 (0.56–1.79) |
| Nordenskjöld et al. 2022(8) | Inpatients with unipolar depression | Propensity score matching and regression analysis | Cardiovascular Death | 0.59 (0.40–0.87) |

HR – Hazard ratio; CI – Confidence Interval

*Acute cardiac events: acute myocardial infarction, cardiac arrest, paroxysmal tachycardia, atrial fibrillation/flutter, and other cardiac arrhythmias

Note – Ahmadi et al. 2016(9) was not included in this table due to methodologic limitations

# Supplemental Table 5 – Studies examining association between ECT and suicide death

| **Study** | **Population** | **Adjustment Method** | **HR (95%CI)** |
| --- | --- | --- | --- |
| Liang et al. 2018(10) | Inpatients with mood disorders | Regression adjustment | 0.80 (0.62–0.94) |
| Peltzman et al. 2020*(11) | Veterans with any psychiatric diagnosis | Propensity score matching and regression adjustment | 1.31 (0.94–1.96) |
| Rhee et al. 2021**(12) | Inpatients with mood disorders ≥65 years | Matching and regression adjustment | 0.56 (0.34–0.92) |
| Ronnqvist et al. 2021**(13) | Inpatients with mood disorders | Propensity score matching | 0.58 (0.40–0.83) |
| Kaster et al. 2022**(14) | Inpatients with mood disorders | Propensity score weighting | 0.39 (0.16–0.91) |

HR – Hazard ratio; CI – Confidence Interval

Note – Ahmadi et al. 2016(9) and Jorgensen et al. 2020(15) were not included in this table due to methodologic limitations

*Reported odds ratio

**90-day outcome

# Supplemental Table 6 – Studies examining association between ECT and all-cause mortality

| **Study** | **Population** | **Adjustment Method** | **HR (95%CI)** |
| --- | --- | --- | --- |
| Liang et al. 2017*(16) | Inpatients with any psychiatric disorder | Regression adjustment | 1.41 (0.90–2.22) |
| Rhee et al. 2021**(12) | Inpatients with mood disorders ≥65 years | Matching and regression adjustment | 0.54 (0.47–0.61) |
| Ronnqvist et al. 2021**(13) | Inpatients with mood disorders | Propensity score matching | 0.43 (0.30–0.61) |
| Watts et al. 2021*(17) | Any psychiatric diagnosis | Propensity score matching | 0.87 (0.79−1.11) |
| Kaster et al. 2022(14) | Inpatients with mood disorders | Propensity score weighting | 0.75 (0.58–0.97) |
| Nordenskjöld et al. 2022(8) | Inpatients with unipolar depression | Propensity score matching and regression analysis | 0.74 (0.61–0.89) |
| Osler et al. 2022(7) | Mood disorder –physical health comorbidity | Regression analysis | 0.71 (0.66–0.77) |
| Osler et al. 2022(7) | Mood disorder – no physical health comorbidity | Regression analysis | 0.62 (0.55–0.69) |
| Hedna et al. 2024**(18) | Inpatients with mood disorders ≥75 years | Propensity score matching and regression adjustment | 0.35 (0.23–0.52) |
| Kirov et al. 2025(4) | Inpatients with mood disorders | Regression adjustment | 0.81 (0.75–0.88) |

HR – Hazard ratio; CI – Confidence Interval

Note – Ahmadi et al. 2016(9) and Jorgensen et al. 2020(15) were not included in this table due to methodologic limitations

*Reported odds ratio

**90-day outcome

# Supplemental Table 7 – Methodologic Limitations of Excluded Studies

## Ahmadi et al. 2016

| **Methodologic Consideration** | **Resulting Bias** |
| --- | --- |
| Controls selected from amongst those without MDD and PTSD | Results in confounding by indication due to presence of psychiatric illness |
| No index time defined for survival analysis | Risk of immortal time bias particularly due to the need to survive until receipt of the ECT procedure |
| Kaplan-Meier survival curves generated to compare risk of suicide | Does not allow for confounder adjustment resulting in confounding by indication |
| Survival analyses report relative risk | Survival analyses, when analyzed using a Cox proportional hazards model report a hazard ratio |
| Analyses did not account for matched nature of sample | The analytic sample does not meet the independent and identically distributed assumption required for standard analytic models |
| Covariates included age, sex, hypertension, dyslipidemia, diabetes mellitus, family history of coronary artery disease, and smoking status | Limited number of covariates results in greater risk of confounding by indication |

## Liang et al. 2018

| **Methodologic Consideration** | **Resulting Bias** |
| --- | --- |
| Controls required to have ≥3 psychiatric hospitalizations and no similar criteria for ECT-exposed individuals | Incomplete confounding adjustment for psychiatric illness severity, which is a critical confounder |
| No index time defined for survival analysis, particularly for control group | Risk of immortal time bias particularly due to the need to survive until receipt of the ECT procedure |
| Regression model includes institutional variables which violates independence assumption and introduces clustering (e.g., within institutions) | Results in incorrectly small standard errors and resulting narrowed confidence intervals |
| Limited number of patient-level confounders included – consisting of age, sex, income, and ‘catastrophic illness’ | This significantly increases the risk of confounding by indication as the differences between ECT and non-ECT controls is inadequately accounted for |

## Jorgensen et al. 2020

| **Methodologic Consideration** | **Resulting Bias** |
| --- | --- |
| Baseline characteristics of individuals not receiving ECT not included in the manuscript reporting | Unable to assess the degree of confounding present in both exposure groups and whether analytic approach was appropriate or successful |
| Individuals with diagnosis of mild depression were included in the cohort | ECT is not clinically indicated for mild depression. Inclusion of these individuals is likely to result in information bias (i.e., data coding error) |
| ECT entered model as a time-varying exposure | Given illness severity is only included in model at baseline, ECT at a later time point is likely influenced by illness severity at that time (i.e., time-varying confounding). Standard analytic models result cannot properly account for time-varying confounding. |
| Covariates assessed at baseline | Given that ECT exposure entered model as time-varying covariate, there is likely to also be unobserved confounding within the regression models that would be highly correlated with depression severity given that there was a relationship between illness severity and time to ECT (Mild depression: 172 days until first ECT vs Severe with psychotic features: 13 days until first ECT)/ |

# Supplemental Table 8 – Study characteristics

| **Study** | **Sample** | **Country** | **Study Design** | **Sample Size** | **Exposed (N)** | **Unexposed (N)** | **Outcome** | **Follow-up** | **Adjustment** | **Covariates** |
| --- | --- | --- | --- | --- | --- | --- | --- | --- | --- | --- |
| Osler et al. 2018 | Patients aged ≥10 years with first-time hospital contact for affective disorder (ICD-10 codes F30.0-F39.9) | Denmark | Cohort study | 168,015 | 5,901 | 5,901 | Incident dementia | From first affective disorder until dementia, death, emigration or end of follow up -Median 4.9 years (IQR 2.4-7.8) | Propensity score matching (1:1 nearest neighbor) followed by Cox regression analysis | Sex, age, education, depression subtype, history of stroke/schizophrenia/alcohol or substance abuse, prescriptions of tricyclic antidepressants/other antidepressants/lithium/antipsychotic medication |
| Chu et al. 2018 | Patients diagnosed with schizophrenia, bipolar disorder and MDD | Taiwan | Matched cohort study | 3,976 | 994 | 2,982 | Incident dementia | From index date until dementia diagnosis, withdrawal from NHI or end of study period (Up to 10 years) | Exact matching and Fine and Gray competing risk model | Age, sex, geographic area of residence, urbanization level of residence, insurance premiums, the Charlson Comorbidity Index. |
| Hjerrild et al. 2021 | In patients diagnosed with affective disorder | Denmark | Matched cohort study | 14,994 | 4,998 | 9,996 | Incident dementia | Median years: 16.9 (ECT), 16.0 (non-ECT), 19.6 (background cohort); maximum follow-up 34 years | Exact matching (1:3 and 1:100 ratios); multivariable Cox proportional hazards regression adjusted for covariates | Number of previous psychiatric admissions, somatic and psychiatric comorbidities increasing risk of dementia |
| Kirov et al. 2025 | Patients hospitalized for affective disorder | Wales, UK | Cohort Study | 110,774 | 1,010 | 109,764 | Incident dementia | Mean: 24.5 years (follow up), 12 years (after first ECT) | Cox proportional hazards, Fine-Gray regression methods | Female sex, Welsh Index of Multiple Deprivation, Charlson Comorbidity Index, affective disorder hospitalization age, number of hospitalizations for affective disorders, alcohol abuse |
| Hsieh et al. 2019 | Patients hospitalized for psychiatric illnesses | Taiwan | Matched cohort study | 24,928 | 6,264 | 18,664 | Incident stoke | From index hospitalization until stroke, death, withdrawal from NHI system, or end of study period | Matched at cohort entry, Cox proportional hazards regression | Age, sex, psychiatric diagnosis, socioeconomic status (enrollee category), level of urbanization, diabetes mellitus, hypertension, hyperlipidemia, coronary heart disease, and Charlson Comorbidity Index score |
| Rozing et al. 2019 | First time hospital contact for affective disorder form 2005-2015 | Denmark | Matched cohort study | 162,595 | 5,781 | 5,781 | Risk of incident or recurrent stroke | Mean follow up: 5.1 years | Propensity score calibration matching (1:1 match), Cox proportional hazard regression, and Fine Gray risk regression matched analysis | Gender, age, level of education, comorbid alcohol misuse, hypertension, coronary heart disease, obesity and diabetes |
| Osler et al. 2022 | Patients with affective disorder | Denmark | Cohort study | 174,495 | 6,943 | 167,552 | Mortality and acute somatic events | From study entry (first affective disorder diagnosis/ECT) until outcome, emigration, or end of follow-up; median follow-up 6.7 years | Cox proportional hazard regression models | Age, sex, marital status, highest achieved educational level, diagnostic subtype of affective disorder, number of chronic somatic comorbidities, and psychopharmacological treatment |
| Nordenskjöld et al. 2022 | Inpatients with moderate or severe unipolar depression | Sweden | Cohort study | 10,952 | 5,476 | 5,476 | Major adverse cardiovascular events (MACE) | 1 Year after admission | Propensity score matching (1:1) and Cox proportional hazards regression | Age, sex, education level, previously diagnosed somatic comorbidities and previously prescribed medications |
| Ahmadi et al. 2016 | MDD and comorbid PTSD patients | United States | Cohort study | 3,485 | 92 | 3,393 | All-cause mortality, cardiovascular mortality and suicide | Medium: 8 years | Matching and Cox regression analysis | age, gender, hypertension, hyperlipidemia, diabetes mellitus, family history of coronary artery disease, smoking status; antidepressant therapy additionally included in suicide models |
| Liang et al. 2018 | Inpatients with unipolar or bipolar disorder | Taiwan | Cohort study | 2,435 | 487 | 1948 | Suicide death | mean follow up, years: 4.4 (ECT group), 2.76 (non-ECT group) | Cox regression models | Age, sex, diagnosis (unipolar vs bipolar), catastrophic illness, Charlson Comorbidity Index, low-income household status, length of hospital stay, hospital level, urbanization level |
| Peltzman et al. 2020 | VHA users with recent inpatients or outpatient mental health encounters | United States | Cohort study | 73,179 | 14,810 | 58,369 | Suicide death | Not reported; outcome defined as suicide within 1 year | Propensity score matching and logistic regression using generalized estimating equation (GEE) | Age, sex, geographic region, medical inpatient stay, emergency department visit, psychiatric diagnoses (including depression, anxiety disorder, PTSD, psychotic disorders, dementia), chronic pain, Charlson Comorbidity Index, and medication use (antidepressants, antipsychotics, benzodiazepines, opioids, mirtazapine, and zolpidem). |
| Rhee et al. 2021 | Psychiatric inpatients aged 65 or older | United States | Matched cohort study | 41,620 | 10,460 | 31,160 | All-cause mortality and suicide death | Outcomes assessed within 30 days and up to 1 year after discharge | Exact matching (1:3) and multivariable Cox proportional hazards regression | Race/ethnicity, rural–urban continuum code, calendar year of hospitalization, median household income of ZIP code, and psychotropic medications prescribed in the year prior to index hospitalization |
| Ronnqvist et al. 2021 | Inpatients with moderate, or severe depression or severe depression with psychosis | Sweden | Cohort study | 11,050 | 5,525 | 5,525 | Suicide death | 3 and 12 months follow up | Exact matching (1:1) followed by propensity score matching and Cox regression | Age, marital status, living alone, employment status, patient education level, parental education level, family history of mental disorder, family history of suicide, compulsory psychiatric treatment, anxiety disorder, personality disorder, alcohol use disorder, substance use disorder, diabetes, obstructive airway disease, ischemic heart disease, heart failure, stroke, cancer, spinal disease, prior suicide attempt by poisoning, prior suicide attempt by other methods, antidepressant use, lithium use |
| Kaster et al. 2022 | Inpatients with bipolar and unipolar depression | Canada | Cohort study | 67,327 | 4,982 | 62,345 | Suicide death | 1 year | Propensity score weighting (weighted by the odds) | >100 potential confounders from a broad range of sociodemographic, clinical, psychiatric and functional symptoms, and health service utilization characteristics |
| Jorgensen et al. 2020 | Patients with first-time hospital contact due to single or recurrent depression | Denmark | Cohort study | 92,895 | 5,004 | 87,891 | Re-hospitalization for depression, suicide attempts, suicide, all-cause mortality | From first depression diagnosis (2005–2015) until date of first ECT, emigration, death, or 31 October 2016 (maximum ≈11.3 years) | Cox proportional hazard regression models | Age, sex, education, marital status, patient type (inpatient vs outpatient), comorbid alcohol abuse, personality disorder, prior stroke, prior suicide attempt, psychotropic medication use, number of prior hospitalizations |
| Liang et al. 2017 | Inpatients with psychiatric conditions | Taiwan | Cohort study | 828,899 | 1,571 | 827,328 | In hospital mortality and ECT application | N/A | Multivariable generalized estimating equations (GEE) | Age, sex, surgery, catastrophic illness, Charlson Comorbidity Index, low-income household status, length of hospital stay, hospital level, urbanization level |
| Watts et al. 2021 | Veterans Affair patients from 2000-2017 | United States | Cohort study | 15,194 | 5,097 | 10,097 | All-cause mortality | 7 days, 30 days and 1 year after index ECT treatment or inpatient discharge | Propensity score matching (1:2 nearest neighbor) and logistic regression | Age, sex, race, geographic region, psychiatric diagnoses, prior suicide attempt, medical comorbidity burden (Charlson Comorbidity Index), pharmacological treatments, and health service use variables (psychiatric inpatient admission, medical inpatient admission, and emergency department use in the year prior to the index date). |
| Hedna et al. 2024 | Inpatients aged ≥75 with moderate to severe depression | Sweden | Cohort study | 6,259 | 1,802 | 4,457 | Suicidal behavior (fatal and non fatal) and all-cause mortality | 1 year after discharge | Exact and propensity score nearest neighbor matching (1:3) and logistic regression | Age, sex, concomitant psychiatric disorder, previous episode of self-harm, Charlson Comorbidity Index , use of psychoactive medications (lithium, antidepressants, hypnotics and anxiolytics) |

# Supplemental Figure 1 – PRISMA Diagram

# Supplemental References

1. Osler M, Rozing MP, Christensen GT, Andersen PK, Jørgensen MB. Electroconvulsive therapy and risk of dementia in patients with affective disorders: a cohort study. Lancet Psychiatry. 2018 Apr;5(4):348–56.

2. Chu CW, Chien WC, Chung CH, Chao PC, Chang HA, Kao YC, et al. Electroconvulsive therapy and risk of dementia—a nationwide cohort study in Taiwan. Frontiers in psychiatry. 2018;9:397.

3. Hjerrild S, Kahlert J, Buchholtz PE, Rosenberg R, Videbech P. Long-term risk of developing dementia after electroconvulsive therapy for affective disorders. The journal of ECT. 2021;37(4):250–5.

4. Kirov G, Simmonds E, Kaster T, Escott-Price V. Risk of dementia after electroconvulsive therapy: a cohort study on the population of Wales. Acta Psychiatrica Scandinavica. 2025;

5. Hsieh KY, Tsai KY, Chou FHC, Chou YM. Reduced risk of stroke among psychiatric patients receiving ECT: a population-based cohort study in Taiwan. Psychiatry Research. 2019;276:107–11.

6. Rozing MP, Jørgensen MB, Osler M. Electroconvulsive therapy and later stroke in patients with affective disorders. The British Journal of Psychiatry. 2019 Mar;214(3):168–70.

7. Osler M, Rozing MP, Jorgensen MB, Jorgensen A. Mortality and acute somatic events following electroconvulsive therapy in patients with pre-existing somatic comorbidity–a register-based nationwide Danish cohort study. The World Journal of Biological Psychiatry. 2022;23(4):318–26.

8. Nordenskjöld A, Güney P, Nordenskjöld AM. Major adverse cardiovascular events following electroconvulsive therapy in depression: a register-based nationwide Swedish cohort study with 1-year follow-up. Journal of Affective Disorders. 2022;296:298–304.

9. Ahmadi N, Moss L, Simon E, Nemeroff CB, Atre-Vaidya N. Efficacy and long-term clinical outcome of comorbid posttraumatic stress disorder and major depressive disorder after electroconvulsive therapy. Depression and Anxiety. 2016 July;33(7):640–7.

10. Liang CS, Chung CH, Ho PS, Tsai CK, Chien WC. Superior anti-suicidal effects of electroconvulsive therapy in unipolar disorder and bipolar depression. Bipolar Disord. 2018 Sept;20(6):539–46.

11. Peltzman T, Shiner B, Watts BV. Effects of Electroconvulsive Therapy on Short-Term Suicide Mortality in a Risk-Matched Patient Population. J ECT. 2020 Sept;36(3):187–92.

12. Rhee TG, Sint K, Olfson M, Gerhard T, H Busch S, Wilkinson ST. Association of ECT With Risks of All-Cause Mortality and Suicide in Older Medicare Patients. Am J Psychiatry. 2021 Dec;178(12):1089–97.

13. Rönnqvist I, Nilsson FK, Nordenskjöld A. Electroconvulsive Therapy and the Risk of Suicide in Hospitalized Patients With Major Depressive Disorder. JAMA Netw Open. 2021 July 1;4(7):e2116589.

14. Kaster TS, Blumberger DM, Gomes T, Sutradhar R, Wijeysundera DN, Vigod SN. Risk of suicide death following electroconvulsive therapy treatment for depression: a propensity score-weighted, retrospective cohort study in Canada. Lancet Psychiatry. 2022 June;9(6):435–46.

15. Jørgensen MB, Rozing MP, Kellner CH, Osler M. Electroconvulsive therapy, depression severity and mortality: Data from the Danish National Patient Registry. Journal of psychopharmacology (Oxford, England). 2020 Jan;269881119895518.

16. Liang CS, Chung CH, Tsai CK, Chien WC. In-hospital mortality among electroconvulsive therapy recipients: a 17-year nationwide population-based retrospective study. European Psychiatry. 2017;42:29–35.

17. Watts BV, Peltzman T, Shiner B. Mortality after electroconvulsive therapy. Br J Psychiatry. 2021 Nov;219(5):588–93.

18. Hedna K, Jonson M, Sigström R, Levinsson A, Nordenskjöld A, Waern M. Suicidal behavior and all-cause mortality in depressed older adults aged 75+ treated with electroconvulsive therapy: A Swedish register-based comparison study. International journal of geriatric psychiatry. 2024;39(5):e6102.
